# Efficacy of galactose and adalimumab in patients with resistant focal segmental glomerulosclerosis: report of the font clinical trial group

**DOI:** 10.1186/s12882-015-0094-5

**Published:** 2015-07-22

**Authors:** Howard Trachtman, Suzanne Vento, Emily Herreshoff, Milena Radeva, Jennifer Gassman, Daniel T. Stein, Virginia J. Savin, Mukut Sharma, Jochen Reiser, Changli Wei, Michael Somers, Tarak Srivastava, Debbie S. Gipson

**Affiliations:** NYU Langone Medical Center, CTSI, Room #110, 227 E 30th Street, New York, NY USA; University of Michigan, Ann Arbor, MI USA; Cleveland Clinic Foundation, Cleveland, OH USA; Albert Einstein College of Medicine, Bronx, NY USA; Kansas City Veteran’s Administration Medical Center, Kansas City, MO USA; Rush University Medical Center, Chicago, IL USA; Boston Children’s Medical Center, Boston, MA USA; Children’s Mercy Hospital, Kansas City, MO USA

**Keywords:** FSGS, Galactose, Adalimumab, Renoprotective, Antifibrotic, Permeability factors

## Abstract

**Background:**

Patients with resistant focal segmental glomerulosclerosis (FSGS) who are unresponsive to corticosteroids and other immunosuppressive agents are at very high risk of progression to end stage kidney disease. In the absence of curative treatment, current therapy centers on renoprotective interventions that reduce proteinuria and fibrosis. The FONT (Novel Therapies for Resistant FSGS) Phase II clinical trial (NCT00814255, Registration date December 22, 2008) was designed to assess the efficacy of adalimumab and galactose compared to standard medical therapy which was comprised of lisinopril, losartan, and atorvastatin.

**Methods:**

Key eligibility criteria were biopsy confirmed primary FSGS or documentation of a causative genetic mutation, urine protein:creatinine ratio >1.0 g/g, and estimated glomerular filtration rate (eGFR) >40 ml/min/1.73 m^2^. The experimental treatments – adalimumab, galactose, standard medical therapy-- were administered for 26 weeks. The primary endpoint was a 50 % reduction in proteinuria with stable eGFR.

**Results:**

Thirty-two subjects were screened and 21 were assigned to one of the three study arms. While none of the adalimumab-treated subjects achieved the primary outcome, 2 subjects in the galactose and 2 in the standard medical therapy arm had a 50 % reduction in proteinuria without a decline in eGFR. The proteinuria response did not correlate with serial changes in the serum glomerular permeability activity measured by the P_alb_ assay or soluble urokinase plasminogen activator receptor (suPAR). There were no serious adverse effects related to treatments in the study.

**Conclusions:**

Recruitment into this trial that addressed patients with resistant FSGS fell short of the enrollment goal. Our findings suggest that future studies of novel therapies for rare glomerular diseases such as FSGS may benefit from enrollment of patients earlier in the course of their disease. In addition, better identification of patients who are likely to respond to a new treatment based on biomarkers suggesting involvement of the disease pathway targeted by the experimental agent may reduce the required sample size and increase the likelihood of a favorable outcome.

**Electronic supplementary material:**

The online version of this article (doi:10.1186/s12882-015-0094-5) contains supplementary material, which is available to authorized users.

## Background

Focal segmental glomerulosclerosis (FSGS) is an important cause of nephrotic syndrome in children and adults and accounts for a sizable number of patients who develop end stage kidney disease (ESKD) [1]. FSGS can be primary, genetic, or secondary to a wide range of clinical conditions including obesity, HIV infection, medications, or reflux nephropathy [2, 3]. Regardless of the underlying etiology, podocyte dysfunction and loss are considered the pivotal events in the pathogenesis of the disease [4]. Current therapy involves administration of corticosteroids as first-line treatment. Administration of an agent that blocks the renin-angiotensin-aldosterone axis to lower proteinuria is standard of care in patients with primary or secondary FSGS. In those who fail to respond to steroids, calcineurin inhibitors are the next step in treatment. However, a significant number of patients with FSGS are resistant to corticosteroids and other immunosuppressive medications [5]. In the recently completed FSGS Clinical Trial, fewer than half of the patients responded to either cyclosporine (44 %) or a combination of oral dexamethasone pulses and mycophenolate mofetil (33 %) [6]. Patients who fail to achieve a significant reduction in proteinuria after treatment with currently available therapeutic options are at high risk of progressing to ESKD [7, 8]. Moreover, in those who require a kidney transplant, nearly 25 % manifest recurrent disease in their renal allograft [9]. These findings underscore the pressing need to develop new treatments for primary idiopathic or genetic FSGS that are safe and well tolerated.

The FONT (Novel Therapies for Resistant FSGS) study was a combined Phase I/II clinical trial designed to test new treatments for patients with refractory FSGS. In the absence of a defined molecular mechanism of disease, the overall objective was to assess antifibrotic agents that would decrease proteinuria and reduce glomerular sclerosis progression. Based on the preclinical and clinical findings implicating the tumor necrosis factor-α (TNF) and peroxisome proliferator activator-γ (PPARγ) pathways in FSGS, the trial was originally designed to compare standard conservative therapy (SCT) *versus* SCT plus adalimumab *versus* SCT plus rosiglitazone [10]. Because of patient concerns about potential adverse cardiovascular consequences of rosiglitazone [11], oral galactose supplementation was substituted for rosiglitazone. This replacement was made based on pre-clinical and clinical data indicating that galactose neutralizes the activity of a circulating FSGS permeability factor [12, 13]. This report summarizes the clinical outcomes of the FONT trial and highlights difficulties that are encountered in the performance of randomized clinical trials in nephrology.

## Methods

The FONT II trial was a Phase I/II open-label RCT designed to select treatments that are worthy of further study in randomized Phase III studies. Enrollment into the study began in July 2009 and was closed in February 2013.

### Inclusion and exclusion criteria

The requirements for enrollment into the FONT study have been described in full detail in a previous report [10]. The key inclusion criteria were: (1) primary FSGS confirmed by renal biopsy or by documented history of a disease-causing mutation [3]; (2) failure to respond to prior therapy with corticosteroids and at least one other immunosuppressive medication; (3) age 1–50 years at onset of proteinuria and 1–51 years at time of randomization; (4) estimated GFR ≥40 mL/min/1.73 m^2^; (5) Urine protein:creatinine ratio (Up/c) > 1.0 g/g on first morning void and (5) no immunosuppressive therapy for at least 30 days except low dose prednisone. Testing for podocyte gene mutations was not required prior to enrollment in the FONT II trial because renal fibrosis is an intrinsic feature of primary FSGS, regardless of whether or not there is a defined genetic mutation.

### Screening and run in phase

In this phase, subjects were placed on the maximal tolerated doses of lisinopril, losartan, and atorvastatin, based on reports of adverse effects and measurements of blood pressure, serum K^+^, creatinine, liver enzymes, and cholesterol concentrations. The doses of the angiotensin converting enzyme inhibitor (ACEi)/angiotensin receptor blocker (ARB) treatment had to be stable for a minimum of 2 weeks prior to randomization. No specific recommendations were made regarding daily dietary sodium or protein intake and this was left to the discretion of the site investigator.

### Study medications

All subjects received standard conservative medical therapy consisting of a combination of the following three agents: 1) lisinopril: 2) losartan; and 3) atorvastatin. The maximum target doses for subjects weighing <40 kg were: lisinopril 10 mg, losartan 25 mg, and atorvastatin 10 mg. For subjects weighing >40 kg, the maximal doses were: lisinopril 20 mg, losartan 50 mg, and atorvastatin 20 mg. The following novel therapies were administered for 6 months:

### Adalimumab *(Humira®, TNF antibody)*

The dose of adalimumab was 24 mg/m^2^ (maximum 40 mg/dose) every other week as a subcutaneous injection for the entire treatment period. Although the pharmacokinetics (PK) data from the FONT-I Study indicated enhanced clearance of adalimumab in subjects with FSGS and nephrotic-range proteinuria, the dose was not increased above the standard dosing regimen for other FDA approved indications to minimize the adverse event risk [14].

*Galactose (Ferro Pfanstiehl, Waukegan, IL)*, 0.2 g/kg per dose was administered orally twice a day, dissolved in 15–30 ml of water and ingested 15–30 min before breakfast and dinner. The maximum single dose was 15 g.

One subject was randomized to rosiglitazone treatment prior to this arm being dropped and replaced with galactose. This subject was not included in the analysis. The use of the full array of experimental therapies in the FONT Phase II Trial was authorized by the FDA (IND # 100,037).

### Primary and secondary outcomes

The co-primary endpoints included:

1. Reduction in proteinuria at 6 months by ≥ 50 % of the value at the time of screening, AND
2. eGFR at 6 months ≥ 75 % of the value at the time of randomization in those with an initial eGFR <75 mL/min/1.73 m^2^ or eGFR persistently ≥75 mL/min/1.73 m^2^ in those whose renal function was ≥75 mL/min/1.73 m^2^ at the time of randomization

The secondary endpoints were: (1) adverse effect profile; (2) percent change in proteinuria (evaluated as continuous variable); (3) change in eGFR, (4) time to halving of eGFR and ESKD

### Study management

**Fig. 1:**
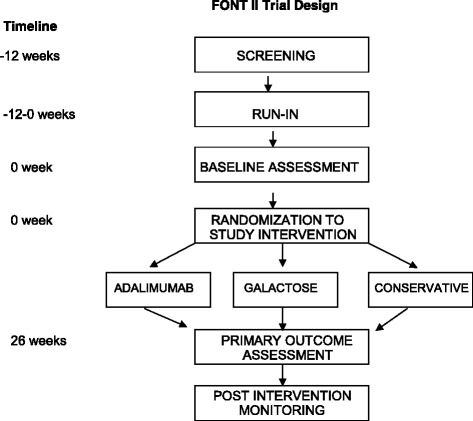
Schematic summary of study design including the various phases of the trial and the three treatment arms

Figure 1 outlines the course of the Phase II trial. Subjects were evaluated after 2, 8, 16, and 26 weeks of the assigned experimental treatment. A follow-up evaluation was performed at 1 month, 3 months, and 6 months after discontinuation of the novel therapy, and then every 6 months until the end of the funding period. At each visit, blood was obtained for determination of CBC and a comprehensive metabolic profile and a first morning urine sample was obtained for measurement of Up/c. P_alb_, a measurement of the capacity of serum to impair the filtration barrier to albumin, was determined at baseline in all subjects and after 8 and 26 weeks in subjects assigned to the galactose arm. The serum galactose concentration was also measured at baseline and after 26 weeks of treatment in those who were treated with galactose.

### Study mandated stop points

Stop points for novel therapy included: 1. >50 % decline from baseline eGFR and <60 mL/min/1.73 m^2^ or a final level <20 mL/min/1.73 m^2^; 2. ESKD, i.e., initiation of dialysis or receipt of a renal transplant; 3. Serious adverse event (SAE), i.e. grade 4 CTC toxicity, version 4 (http://evs.nci.nih.gov/ftp1/CTCAE/CTCAE_4.03_2010-06-14_QuickReference_5×7.pdf); 4. Increase in ALT/AST to >2.5×the upper limit of normal; 5. Onset of congestive heart failure or a myocardial infarction; 6. Clinical onset of SLE and/or positive ANA ≥ 1:160; 7. Serious infection including sepsis; 8. Malignancy; and 9. Pregnancy.

### Laboratory methods

All routine serum and urine biochemical tests were performed in a central laboratory (Spectra East, Rockleigh, NJ).

Galactose was measured in the serum by GC/MS via modification of a published procedure [15]. In brief, 50 μL of 100 μg/mL U-13C galactose (Cambridge Isotope Labs) internal standard prepared in deionized water was added to 200 μL of serum. All other reagents were obtained from Sigma unless otherwise specified. In order to deplete glucose in the samples, 100 μL 2.5 M potassium bicarbonate, 50 μL 2.5 M phosphate buffer pH 6.5, 25 μL 300 U/μL glucose oxidase in 0.1 M potassium phosphate buffer pH 7 and 25 μL 50U/μL Catalase in 0.05 M phosphate buffer pH 7, were added and then incubated for 2 h at 37 °C with mixing. 200 μL each of 0.3 N zinc sulfate and 0.3 N barium hydroxide were added, the samples were vortexed and left on ice for 30 min. The samples were centrifuged at 3200 rpm for 10 min.

Prior to derivatization, the sample supernatants were deionized using cation exchange resin (AG50W-×8 hydrogen form) and anion exchange resin (AG 1-×8, formate form) (Biorad). Samples were eluted with deionized water and then dried. Galactose was derivatized to its acetate derivative for GC/MS analysis. 100 μL 20 mg/mL hydroxylamine hydrochloride/pyridine was added to the dried sample and heated at 70 °C for 30 min. 150 μL acetic anhydride was added with continued heat at 70 °C for another 30 min. The samples were transfer to vials with inserts for GC/MS analysis.

GC/MS analysis of galactose was done with an Agilent 6890 N Gas Chromatograph equipped with a 7683 Auto sampler and 5973 Mass Spectrometer and a DB 5 ms capillary column of 40 m × 0.18 mm × 0.18 μm (Agilent Technologies). Helium was used as carrier gas with a constant flow rate of 1.2 mL/min. Inlet temperature held at 280 °C. The initial oven temperature held at 180 °C for 30 sec and temperature ramps 50^0^/min to 220^0^ hold for 30 sec followed by 50^0^/min to 300 °C hold for 2 min. 1 μL of derivatized sample was injected in 5:1 split mode.

Mass spectrometer transfer line temperature held at 280 °C and operated in electron impact ionization mode at 70 eV. Selective Ion Monitoring (SIM) was used for fragments 314.1 and 319.1, 30 msec dwell time for each. Galactose concentrations were calculated from relative peak areas of ions 314.1/319.1.

P_alb_ was determined by the change in glomerular volume after exposure to media with a reduced albumin concentration and oncotic pressure in accord with previously published methods [16]. Serum suPAR was measured with the Quantikine Human suPAR Immunoassay (R&D Systems). Podocyte β3 integrin activity was determined by AP5 immunostaining of differentiated human podocytes *in vitro*, accord with previously published method [17].

### Statistical methods for analysis of FONT 2 outcome

Continuous data are reported as mean ± SD and in select instances the median (25, 75 percentile). As noted in the study design paper [10], when FONT II was initiated, the primary analysis of outcome was based on a hybrid Phase II design [18] with the study conducted in two phases. The first phase of the study would have ended when 17 participants had been randomized to each treatment arm. At that point, a ranking/selection comparison [19, 20] would have been done between the treatment groups and a minimum rate of success criterion would have been applied to each treatment group. After that, successful treatments would have moved forward to a second phase of investigation. An experimental therapy in which at least 2 out of the first 17 patients responded would have been advanced to the full Phase II assessment. A total of 42 subjects would have been assigned to each group (15 additional participants would have been enrolled in the galactose arm to ensure that 42 had a P_alb_ > 0.5). However, because of limited enrollment, the hybrid Phase II design could not be followed. Instead, the results were analyzed using the Freeman-Halton extension of Fisher’s exact test.

### Ethics of human subject research

The study protocol, design and consent forms were approved by Institutional Review Board (IRB) at each participating site. This included the Institutional Review Board of North Shore/LIJ-Cohen Children’s Medical Center, Cincinnati Children’s Medical Center, Boston Children’s Hospital, Nationwide Children’s Hospital, Doernbecker Children’s Hospital, Carolinas Medical Center, University of Alberta-Stollery Children’s Hospital, Texas Tech University, Children’s Mercy Hospital, University of Kansas, University of Michigan, Columbia University-College of Physician’s and Surgeons, NYU School of Medicine-NYU Langone Medical Center, and University of Miami. Written informed consent was obtained from all participants or, where participants were children, from a parent or guardian. Assent was also obtained from children and adolescents in accord with guidelines at each site. The trial was authorized by the FDA under IND number 103,147. It was listed at ClinicalTrials.gov with the identifier NCT00814255 and the registration date was December 22, 2008.

## Results

### Subject characteristics

**Fig. 2:**
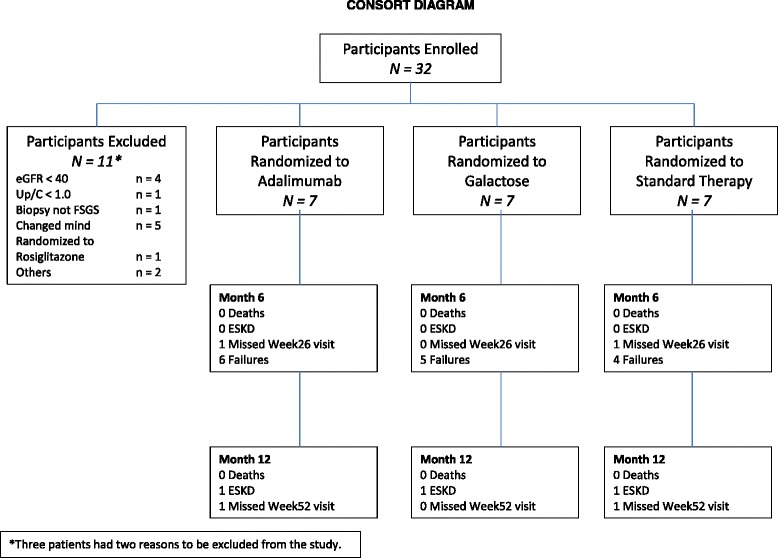
CONSORT diagram summarizing the number of subjects who were screened, enrolled, and randomized to the three treatment arms. In addition, the subject outcomes are provided at the completion of the 6 month Treatment Period and after 6 and 12 months of observation following the Treatment Period

Thirty-two subjects consented and enrolled in the study. However, only 21 subjects were randomized to one of the treatment arms (Fig. 2). The reasons that subjects withdrew or were dropped prior to randomization are detailed in the CONSORT diagram. The duration of the screening period was 1.8 (1.0, 2.3) (median (25, 75th percentile) months. Subjects were followed for 1.6 (1.2, 1.7) years after randomization.

The demographic and clinical features at baseline of the subjects who were randomized to one of the experimental treatments are summarized in Table 1.

**Table 1:** Font trial: demographic and clinical features of the subject Cohort

		Age at consent	Age at consent
	Overall (*N* = 21)	<18 years (*N* = 14)	≥18 years (*N* = 7)
	*N* (%) or Median (IQR)	*N* (%) or Median (IQR)	*N* (%) or Median (IQR)
Age at Consent (yr)	14.7 (13.0, 20.8)	13.5 (12.8, 14.7)	28.6 (20.8, 34.0)
Female	12 (57.1 %)	8 (57.1 %)	4 (57.1 %)
Race (self-reported)			
Black or African American	4 (19.0 %)	2 (14.3 %)	2 (28.6 %)
White	12 (57.1 %)	7 (50.0 %)	5 (71.4 %)
More than one race	1 (4.8 %)	1 (7.1 %)	
Unknown	4 (19.0 %)	4 (28.6 %)	
Ethnicity (Self-reported)	7 (33.3 %)	6 (42.9 %)	1 (14.3 %)
Sitting Systolic BP	109 (104, 120)	109 (88.0, 115)	126 (107, 129)
Sitting Diastolic BP	66.0 (60.0, 74.0)	62.5 (54.0, 71.0)	74.0 (68.0, 79.0)
Previous Rx with Cyclosporine	12 (57.1 %)	6 (42.9 %)	6 (85.7 %)
Cyclosporine Rx: Cumulative Exposure (mos)	6.00 (5.50, 12.0)	5.50 (4.00, 12.0)	6.00 (6.00, 12.0)
Previous Rx with Tacrolimus	13 (61.9 %)	8 (57.1 %)	5 (71.4 %)
Tacrolimus Rx: Cumulative Exposure (mos)	7.00 (4.00, 24.0)	9.50 (4.00, 31.0)	6.00 (6.00, 24.0)
Previous Rx with Mycophenolate	11 (52.4 %)	9 (64.3 %)	2 (28.6 %)
Mycophenolate Rx: Cumulative Exposure (mos)	6.00 (4.00, 17.0)	6.00 (5.00, 12.0)	9.50 (1.00, 18.0)
Edema			
None	14 (66.7 %)	9 (64.3 %)	5 (71.4 %)
Pretibial	6 (28.6 %)	4 (28.6 %)	2 (28.6 %)
Ascites	1 (4.8 %)	1 (7.1 %)	0 (0 %)
Serum Albumin (g/dL) at Screening	2.40 (2.10, 3.50)	2.40 (2.05, 3.60)	2.50 (2.10, 3.50)
Total Cholesterol (mg/dL) at Screening	276 (198, 424)	297 (163, 452)	267 (198, 424)
Up/c at Screening	4.93 (3.30, 11.5)	9.28 (3.30, 12.2)	3.41 (3.23, 4.93)
Baseline eGFR (ml/min/1.73 m2)	120 (81.1, 170)	119 (88.2, 221)	120 (71.5, 151)
Duration of Follow up Post Randomization (yr)	1.63 (1.27, 1.74)	1.66 (1.48, 2.03)	1.24 (1.06, 1.70)

Note: Excludes the subject randomized to Rosiglitazone

### Clinical outcomes

**Fig. 3:**
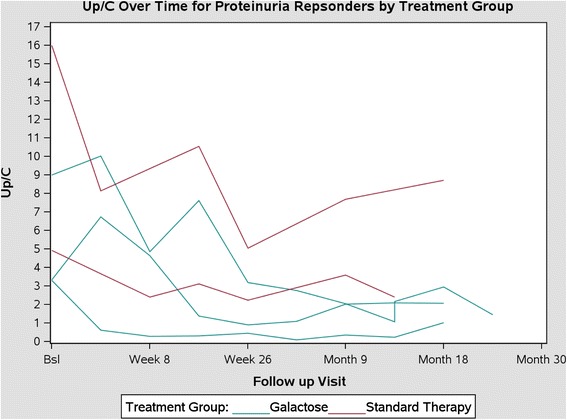
Time course of the response, assessed by the Up/c in a first morning urine sample, in the 5 subjects who achieved a ≥50 % reduction in proteinuria in response to the experimental therapy

The main clinical outcomes for the 21 participants are summarized in Table 2. When examining change in proteinuria alone, none of the subjects assigned to adalimumab therapy achieved a 50 % reduction in proteinuria. In contrast, 3 out of 7 subjects assigned to the galactose arm and 2 out of the 7 subjects assigned standard conservative treatment manifested at least a 50 % decline in proteinuria. The favorable response was sustained for 3–12 months after discontinuation of the galactose but maintenance of all other treatments. In contrast, the effect of standard conservative therapy was not sustained after the completion of the treatment period. The primary outcomes, based on the combined proteinuria and eGFR endpoint, are shown in Table 3. No significant difference in the rates of success was seen (P = 0.48). The change in Up/c over time in the 5 subjects who had a favorable response to the experimental treatment is illustrated in Fig. 3. There were no significant differences in clinical features (age, gender, race) or main laboratory data (eGFR, Up/c, serum albumin concentration) between the 5 subjects who achieved a 50 % reduction in proteinuria and the 16 other participants who had persistent proteinuria that was unresponsive to treatment (Additional file 1: Table S1).

**Table 2:** Font trial: Clinical outcomes

Participant	Up/c month 0	Up/c month 6	% Change Up/c	Up/c responder	eGFR month 0	eGFR month 6	eGFR preservation	Primary outcome met?	Duration (yr) follow-up post randomization
RX = Adalimumab									
1	2.1	12.3	475.0	No	325	41	No	No	2.5
2	6.3	6.6	5.0	No	140	155	Yes	No	2.0
3	14.9	8.4	−43.8	No	88	116	Yes	No	1.6
4	4.7	6.1	31.0	No	120	93	Yes	No	1.7
5^a^,^b^	3.4	.	.	Never received study drug	81	.	Never received study drug	No	0.1
6	2.3	1.6	−30.5	No	37	29	Yes	No	1.5
7	2.1	5.2	142.5	No	58	24	No	No	1.7
RX = Galactose									
8	9.0	3.2	−64.5	Yes	71	41	No	No	2.1
9	3.3	0.9	−72.8	Yes	143	129	Yes	Yes	1.7
10	3.3	0.5	−86.0	Yes	97	91	Yes	Yes	1.6
11	12.2	10.8	−11.4	No	91	63^c^	No	No	1.4
12^a^	49.3	29.9	−39.2	No	63	35	No	No	1.6
13	1.5	1.0	−29.9	No	221	180	Yes	No	1.3
14	6.7	7.0	4.7	No	71	67	Yes	No	1.1
RX = Standard Therapy (lisinopril, losartan, atorvastatin)									
15	4.6	2.8	−40.3	No	276	283	Yes	No	2.1
16	16.0	5.0	−68.5	Yes	235	180	Yes	Yes	1.5
17^a^,^b^	11.6	.	.	Never received study drug	170	.	Never received study drug	No	0.0
18	3.2	2.5	−22.7	No	151	108	Yes	No	1.2
19	4.9	2.2	−54.6	Yes	155	150^c^	Yes	Yes	1.2
20	9.6	5.5	−42.2	No	173	178	Yes	No	1.7
21	11.5	14.2	23.2	No	94	.57^c^	No	No	1.8

Abbreviations: eGFR, estimated glomerular filtration rate; Up/C urine protein:creatinine ratio 1) Excludes the subject randomized to Rosiglitazone; 2) One participant in each arm was neither on ACEi, nor ARB ^a^,Participant did not receive neither ACEi, nor ARB; ^b^, Participant did not start assigned trial therapy; ^c^Participant had eGFR calculated based on testing performed at a local laboratory

**Table 3:** Font trial: outcomes by treatment arm

	Treatment arm			
Outcome	Adalimumab	Galactose	Standard	Total
Success	0	2	2	4
Failure	7	5	5	17
Total	7	7	7	21

The co-primary endpoints included: (1) Reduction in proteinuria at 6 months by ≥ 50 % of the value at the time of screening, AND (2) Estimated GFR (eGFR) at 6 months ≥ 75 % of the value at the time of randomization in those with an initial eGFR <75 mL/min/1.73 m^2^ OR eGFR persistently ≥75 mL/min/1.73 m^2^ in those whose renal function was ≥75 mL/min/1.73 m^2^ at the time of randomization

### Galactose levels

The serum galactose levels prior to treatment, after 8 weeks, and 26 weeks of galactose treatment were 0.23 ± 0.11, 0.68 ± 0.77, and 0.18 ± 0.06 μmol/L, respectively. These values did not differ from each other (*P* = 0.10). The baseline value in 10 subjects who were assigned to either standard conservative therapy or adalimumab treatment was 0.31 ± 0.17 μmol/L, which was similar to the concentration at baseline in the participants who were assigned to galactose therapy.

### Biomarkers – P_alb_ and suPAR

**Fig. 4:**
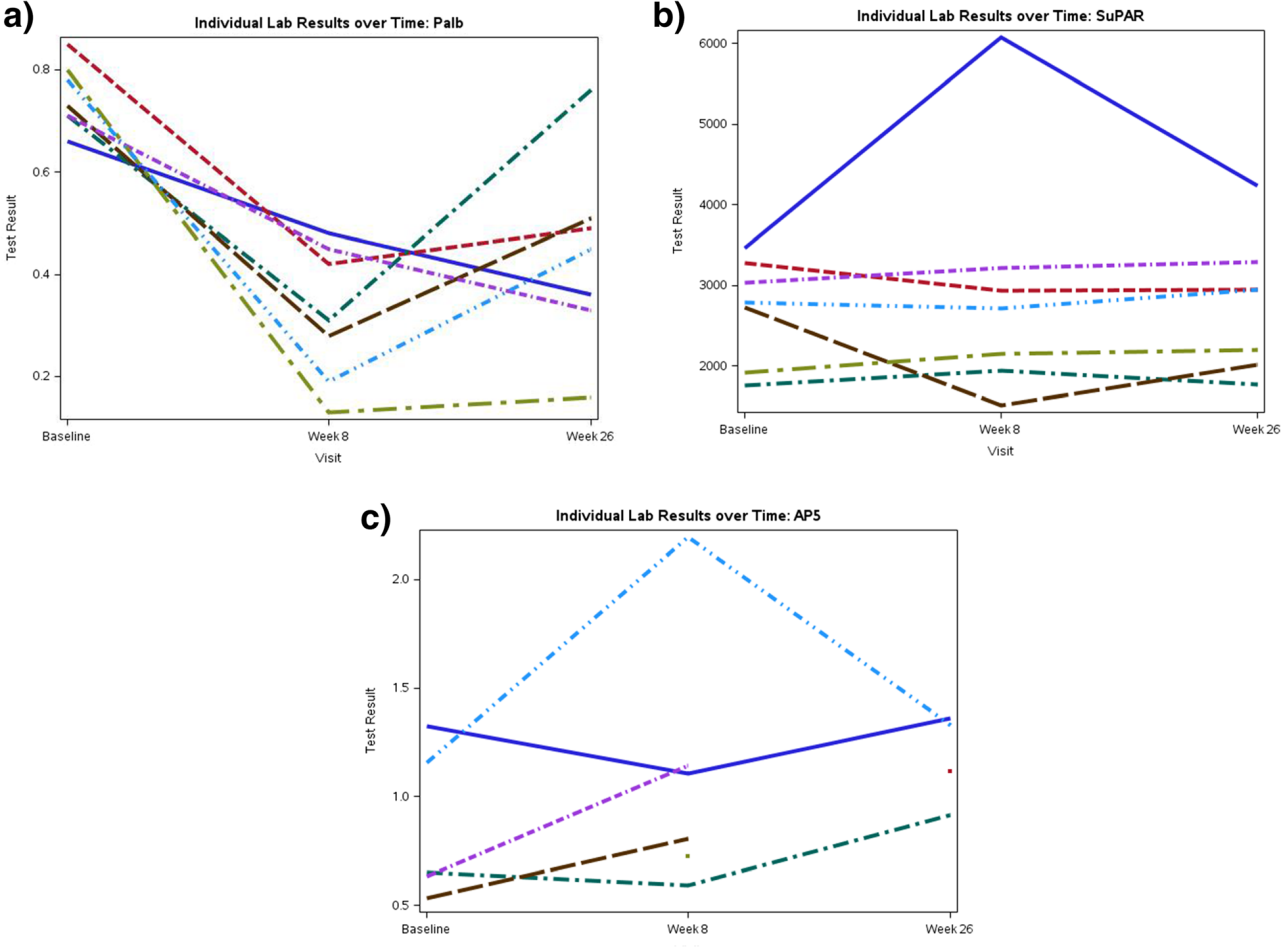
Serial changes at baseline, week 8, and week 26 in the subjects who were assigned the galactose treatment for (**a**) P_alb_; (**b**) suPAR; and (**c**) AP5. Each line represents a single subject

The P_alb_, suPAR, and AP5 values at the onset of the study and after 8 and 26 weeks of galactose treatment are depicted in Fig. 4. At the time of randomization, P_alb_ was ≥ 0.5 (normal P_alb_: <0.2) in all 18 subjects (8 assigned to the galactose [1 child was withdrawn because of an elevated ANA titer in the baseline sample after randomization]), 4 to the adalimumab and 6 to the standard medical treatment arm) in whom the assay was performed. It declined in all 7 subjects who received oral galactose treatment and the value was within the normal range throughout the course of therapy (Fig. 4a). In contrast, the P_alb_ value was unchanged after the 26-week treatment period in 2 subjects who received adalimumab and 2 on standard medical treatment (data not shown). The decline in P_alb_ was significantly different in the galactose *versus* the two treatment groups (*P* = 0.003). The consistent decline in P_alb_ in the subjects assigned to galactose was associated with a 50 % or greater decrease in 3 of 7 subjects (see above). Plasma suPAR levels were initially elevated (>3,000 pg/ml) in 8 out of 22 (36 %) subjects who had measurement performed at baseline. There was no consistent change in circulating suPAR concentration in response to galactose therapy and no consistent relationship between suPAR levels and the change in proteinuria over the 26-week treatment period (Fig. 4b). Similarly, there was no consistent change in AP5 staining of podocytes incubated with subject serum during the course of any treatment and this biomarker did not correlate with the reduction in urinary protein excretion (Fig. 4c). There was no correlation between P_alb_ and suPAR or AP5 staining in the analyzed subjects.

### Adverse outcomes

The serious adverse events that occurred during the study are summarized in Table 4. The other adverse effects are itemized in Table 5. Although most of the subjects did experience at least one averse event during the treatment period, these side effects did not necessitate discontinuation of the assigned experimental treatment.

**Table 4:** Font trial: serious adverse events

Event	Adalimumab (*N* = 7)			Galactose (*N* = 7)			Standard Therapy (*N* = 7)		
	*N* PTs with events	% Rand PTs	*N* Events	N PTs with events	% Rand PTs	*N* events	*N* PTs with events	% Rand PTs	*N* events
Fatal	0	0	0	0	0	0	0	0	0
Immediate Life Threatening	0	0	0	0	0	0	0	0	0
Required Hospitalization	3	42.9	32	1	14.3	9	1	14.3	1
Prolonged Existing Hospitalization	0	0	0	0	0	0	0	0	0
Persistent or Significant Disability/Incapacity	0	0	0	1	14.3	1	0	0	0
Congenital Anomaly / Birth Defect	1	14.3	1	1	14.3	1	0	0	0
Causes Cancer	0	0	0	0	0	0	0	0	0
Overdose of Study Medication	0	0	0	0	0	0	0	0	0

This Table summarizes the serious adverse events that occurred throughout the study period from screening through to the end of the 6 month observation period after the completion of the experimental therapy

**Table 5:** Font trial: adverse events

Symptom category	Adalmumab (*N* = 7)			Galactose (*N* = 7)			Standard Therapy (*N* = 7)		
	*N* PTs with events	% of Rand PTs	*N* events	*N* PTs with events	% of Rand PTs	*N* events	*N* PTs with events	% of Rand PTs	*N* events
Allergy	1	14.3	1	2	28.6	2	1	14.3	1
Anorexia	2	28.6	4	1	14.3	1	0	0	0
CV	0	0	0	1	14.3	1	0	0	0
Cataract	0	0	0	1	14.3	1	0	0	0
Cosmetic	0	0	0	0	0	0	1	14.3	2
Cough	3	42.9	3	2	28.6	3	1	14.3	1
Dehydration	2	28.6	5	1	14.3	5	0	0	0
Dizziness	0	0	0	4	57.1	6	1	14.3	1
Edema	6	85.7	100	5	71.4	57	6	85.7	23
Fatigue	5	71.4	8	2	28.6	5	1	14.3	1
GI	4	57.1	7	2	28.6	6	1	14.3	2
Headache	4	57.1	6	4	57.1	8	1	14.3	1
Hypotension	1	14.3	4	0	0	0	0	0	0
Infection	5	71.4	49	5	71.4	20	4	57.1	10
Miscellaneous	4	57.1	13	4	57.1	7	4	57.1	13
Musculoskeletal	3	42.9	8	1	14.3	2	4	57.1	8
Nausea	2	28.6	2	2	28.6	12	0	0	0
Pain	2	28.6	9	4	57.1	14	3	42.9	3
Renal	2	28.6	8	1	14.3	4	1	14.3	1
Respiratory	1	14.3	2	2	28.6	2	2	28.6	2
Skin	2	28.6	3	3	42.9	14	3	42.9	8
Vomiting	2	28.6	5	2	28.6	5	0	0	0

Number of Participants with Adverse Events Reported Overall After Consent

## Discussion

### General

The only treatment that satisfied the criterion for success in this limited study of novel therapies for patients with resistant FSGS was galactose. Oral administration of this monosaccharide achieved at least a 50 % reduction in proteinuria in 3 out of 7 or 42 % of the treated subjects, 2 of whom maintained a stable eGFR. It is difficult to interpret the response to galactose therapy in response to the serial measurements of the circulating level of the sugar. Although there was no significant change in serum galactose concentration over the course of the study, this is not unexpected because galactose is rapidly metabolized to glucose. In addition, the serum galactose concentration was measured at arbitrary times and we did not standardize blood sampling in relationship to the oral dosing schedule (trough level or fixed interval after ingesting the monosaccharide). However, the consistent decrease in P_alb_ in all subjects who received galactose suggests that all of the subjects adhered to the assigned treatment and is consistent with prior experience with the sugar [12]. There was no evidence of adverse effects related to the use of galactose and future studies of this agent may benefit from a stable formulation that is already prepared ready for use. Our findings are somewhat at variance with the data of Sgambat et al. [21] who administered galactose to 7 pediatric subjects with steroid-resistant nephrotic syndrome (2 with post-transplant FSGS), none of whom achieved a reduction in proteinuria despite a fall in P_alb_. Differences in age, confirmation of the diagnosis of FSGS, by renal biopsy, and inclusion of patients with recurrent disease may account for the modest differences between this negative report and our findings.

It is worth noting that while 0 out of 7 subjects who were assigned to adalimumab in the FONT trial achieved a favorable response to treatment, 4 out of 10 subjects who received adalimumab over 16 weeks in FONT I had at least a 50 % decline in proteinuria [14]. Preliminary data from the NEPTUNE cohort study indicate that there is genomic evidence of TNF activation in a subset of patients with primary FSGS (unpublished observations). It is plausible that future efforts to delineate a molecular signature of TNF activation may be useful in identifying individual patients with FSGS who are more likely to respond to a TNF antagonist. It is becoming increasingly apparent that primary FSGS is a heterogeneous disorder with some cases representing a genetic disorder of the podocyte and others arising from alterations in the integrity of the glomerular barrier due to a circulating factor (see below). It is anticipated that in the future, improved efforts to characterize patients based on their underlying genetic and molecular profiling will facilitate better selection of patients who have a greater likelihood of responding to a targeted therapy. Two out of the 7 subjects who were assigned to conservative medical therapy also achieved a 50 % reduction in proteinuria, which supports the need to include this control arm in any prospective trial of a novel therapeutic agent in patients with resistant FSGS.

There are several features about the FONT study design that are worthy of note. The entry criterion, Up/c > 1, is lower than a cut-off value of 2 used in most studies. As such, it might appear to be less stringent than the usual definition of partial response, namely a 50 % decline in proteinuria from an initial value exceeding 2. The definition used in this trial was an extension of the entry criterion into the FSGS Clinical trial [6]. In fact, the mean Up/c was 4.9 and exceeded 2 in all cases. Moreover, using a cut-off of Up/c > 2 did not alter the results. The study was open label because of a reluctance to implement sham injections in children. However, because the primary outcome involved objective laboratory measurements, it is unlikely that this influenced the outcomes of the trial.

### Permeability biomarkers - P_alb_ and suPAR concentration

Our data suggest that the permeability factors that are defined by the P_alb_ bioassay [22] and the currently available suPAR ELISA kits are distinct entities [17, 23]. While P_alb_ decreased with galactose administration in every subject after 8 weeks, suPAR levels were unchanged throughout the cohort regardless of treatment. In the analyzed samples, there was no correlation between these two potential biomarkers or between either marker and the proteinuria response. Moreover, neither measurement in isolation fully accounted for the persistence or decline in proteinuria in this study. This finding is in contrast to earlier studies which suggested that elevated suPAR levels may be a biomarker for primary FSGS [17, 23]. Recent data suggest that full length suPAR may cause proteinuria in the presence of specific antibodies, e.g. auto-antibodies against CD40, in patients who develop recurrent FSGS after receiving a kidney transplant [24]. A similar 2-hit process may be involved in the pathogenesis of proteinuria in response to other putative circulating factors. We urge caution in interpreting our data about P_alb_ and suPAR in this study because of the limited sample size and the collection of samples fairly late during the course of disease in these subjects with refractory FSGS. Further prospective studies are needed involving patients with all types of glomerular disease including FSGS to determine the prognostic implications of these circulating factors and their role in the pathogenesis of proteinuria and kidney injury.

### Problems with Clinical Trials in FSGS

It is recognized that nephrology ranks near the bottom among medical specialties in the performance and completion of clinical trials [25, 26]. Enrollment into the FSGS Clinical Trial that served as the antecedent to the FONT Trial was below expectations [27]. Poor subject comprehension of the adverse consequences of CKD, the adverse effects of many proposed treatments, and the high incidence of rapid decline in kidney function in patients with refractory proteinuria are among the explanations that have been offered to account for low enrollment into nephrology studies and specifically trials of glomerular diseases. It is likely that all of these factors contributed to the low recruitment into the FONT trial. Problems with subject recruitment underscore the need to create an infrastructure for clinical research and to design pragmatic trials that can be implemented in a timely manner to test potential treatments in patients with rare diseases like FSGS.

Two additional and specific factors may have played a role in limiting recruitment into the FONT Trial. First, by restricting the enrollment to patients who had failed steroids and at least one other immunosuppressive medication, we preselected for those with established disease of several years duration. These patients may be at very high risk of rapid deterioration in kidney function compared to patients evaluated soon after the onset of the disease. In fact, several of the treatment resistant subjects who were offered participation in the study experienced a rapid decline in GFR during the screening period and became ineligible. These observations suggest that it may be prudent to consider clinical trials of novel therapies earlier in the course of glomerular disease with persistent proteinuria. Such patients may have more reversible anatomic changes to glomeruli and the interstitium and their renal function may be more likely to remain stable during the course of the screening evaluation and experimental treatment period. Second, this trial transpired during a period when rosiglitazone and adalimumab were each receiving intense scrutiny because of reports of serious adverse events. Specifically, the FONT trial study was launched almost simultaneously with the publication of a meta-analysis that suggested that rosiglitazone increased the risk of cardiovascular complications in adults receiving the drug for the management of type 2 diabetes [11]. Although the FDA never questioned the rationale for testing thiazolidinediones in patients with resistant FSGS, the ongoing adverse publicity surrounding the use of PPAR-γ agonists dampened patient willingness to be randomized to that arm. As a consequence, we replaced rosiglitazone with galactose, a test agent with no known adverse effects. In addition, a report appeared at nearly the same time linking the adalimumab to the occurrence of serious infections and malignancy [28]. This diminished physician enthusiasm for adalimumab as a treatment arm in FONT even though the adverse consequences of adalimumab were documented in patients who receiving adalimumab in conjunction with other immunosuppressive drugs as treatment for rheumatological diseases. A follow-up report substantially downgraded the level of risk associated with adalimumab [29]. Finally, it is unclear what impact subject perception of a randomization scheme that compared an oral treatment thought to have minimal risk (galactose) to an injectable drug with a well-defined risk profile had on enrollment. Some potential participants expressed concern about a perceived imbalance in risk between the study arms and voiced a strong preference for galactose. They may have declined to participate in the trial without assurance that galactose was the test therapy they would receive. These observations remind us that clinical trials occur in a real world context and enrollment can be seriously impacted by a wide range of extraneous factors, some that are beyond the control of the investigative team and others that can be addressed during protocol development.

## Conclusion

The FONT Trial provides data that must be considered as hypothesis generating rather than hypothesis testing due to the small sample size. Our findings suggest that adalimumab is not a viable agent for further study in patients with resistant FSGS. However, taking into account the combined results of the Phase I (4 out of 10 subjects had a 50 % reduction in proteinuria in response to a 16-week course of treatment [14]) and Phase II studies of adalimumab, additional investigation may be warranted to determine if a subgroup of subjects with TNF associated disease can be identified who would benefit from treatment with inhibitors of the cytokine. Galactose also appears to be of potential value because of proteinuria reduction and preservation of kidney function in some subjects as well as its ease of administration and excellent side effect profile. However, future testing will be necessary and may be strengthened by better delineation of the relationship between P_alb_ and fibrosis and enrollment of patients with earlier disease. Implementation of alternative study designs such as adaptive clinical trials or “n-of-1” trials may be useful to assess response to novel treatments in patients with rare conditions such as FSGS [30, 31]. It is not justified to proceed to a formal Phase II or III trial of either adalimumab or galactose in a non-selected cohort of patients with resistant FSGS based on the findings of the FONT study. The results add to the body of evidence that suggests that future trials of novel agents in FSGS will benefit from a selected cohort approach where patients with a particular mechanism of disease are enrolled into trials of agents that are directed to the active target. The inclusion of a control or standard therapy arm remains mandatory. FSGS includes a constellation of diseases categorized by common pathology features. Standard therapies have approached these diseases as a single entity with the trialing of a series of therapies until a response occurs or futility is reached following multiple drug failures. This FONT trial launched with Phase I in 2008 and Phase II in 2009 and was designed to test novel therapies in individuals with multi-drug resistance. While these treatment resistant patients assuredly need better therapies, a contemporary approach based on molecular profiling of an individual patient may improve the selection of initial target-based therapy. Benefits of a targeted approach may also minimize the cumulative drug related toxicities that accompany sequential therapies observed in these patients. Making the transition to precision medicine will require partnership with patients, clinicians, investigators, and the pharmaceutical and biotechnology industries for target identification, trial design, early phase drug development and drug testing. Finally, a greater willingness on the part of clinicians and patients will be required to engage in the process of discarding drugs with poor risk-benefit profiles while testing promising agents early in the disease course.

## Additional file

Additional file 1: Table S1. Font trial: comparison of proteinuria responder *versus* resistant subjects.

## Abbreviations

ACEi: Angiotensin converting enzyme inhibitor
ARB: Angiotensin receptor blocker
CKD: Chronic kidney disease
ESKD: End stage kidney disease
FONT: Novel Therapies for Resistant FSGS
FSGS: Focal segmental glomerulosclerosis
GFR: Glomerular filtration rate
IRB: Institutional review board
PK: Pharmacokinetic
SAE: Serious adverse event
SCT: Standard conservative therapy
TNF: Tumor necrosis factor-α
Up/c: Urine protein:creatinine ratio
