# Supplementary material for: Efficacy of galactose and adalimumab in patients with resistant focal segmental glomerulosclerosis: report of the font clinical trial group
Source: BMC Nephrol. 2015 Jul 22;16:111. doi: 10.1186/s12882-015-0094-5 (PMC4511259; doi:10.1186/s12882-015-0094-5)
Supplement: Additional file 1: Table S1. — Font trial: comparison of proteinuria responder versus resistant subjects. [file 12882_2015_94_MOESM1_ESM.docx]

**SUPPLEMENTAL TABLE**

**FONT TRIAL: Comparison of Proteinuria Responder versus Resistant Subjects**

|  | Up/c Responders | Up/c Resistant | p-value |
| --- | --- | --- | --- |
|  |  |  |  |
| Age at Consent (yrs) | 16.5 (13.1, 18.1) | 14.6 (13.0, 20.8) | 0.96 |
| Sex |  |  | 0.36 |
| M | 1 (20.0%) | 6 (42.9%) |  |
| F | 4 (80.0%) | 8 (57.1%) |  |
| Race (self-reported) |  |  | 0.93 |
| Black or African American | 1 (20.0%) | 2 (14.3%) |  |
| White | 3 (60.0%) | 8 (57.1%) |  |
| More than one race |  | 1 (7.1%) |  |
| Unknown | 1 (20.0%) | 3 (21.4%) |  |
| Serum Albumin (g/dL) at Screening | 2.50 (2.40, 3.50) | 2.25 (2.00, 3.25) | 0.34 |
| Urine Protein/Creatinine at Screening | 4.93 (3.33, 9.00) | 5.46 (2.34, 11.5) | 0.75 |
| Baseline eGFR (ml/min/1.73 m^2^) | 143 (97.4, 155) | 107 (71.5, 173) | 0.61 |
